# A Systematic Review of Telomere Length and Telomerase Activity in Preeclampsia: Maternal, Placental, and Cord Blood Perspectives

**DOI:** 10.3390/medsci14010100

**Published:** 2026-02-19

**Authors:** Angeliki Gerede, Efthymios Oikonomou, Christos Chatzakis, Sofoklis Stavros, Maria Danavasi, Anastasios Potiris, Ismini Anagnostaki, Theodoros Karampitsakos, Charalampos Theofanakis, Ekaterini Domali, Alexandros Sotiriadis

**Affiliations:** 1 Department of Obstetrics and Gynecology, Democritus University of Thrace, 69100 Alexandroupolis, Greece; eftoikonomou@outlook.com (E.O.); mairidanavasi4800@gmail.com (M.D.); 2 Second Department of Obstetrics and Gynecology, Medical School, Aristotle University of Thessaloniki, 54124 Thessaloniki, Greece; cchatzakis@gmail.com (C.C.); asotir@gmail.com (A.S.); 3 Third Department of Obstetrics and Gynecology, University General Hospital “ATTIKON”, Medical School, National and Kapodistrian University of Athens, 12462 Athens, Greece; sfstavrou@med.uoa.gr (S.S.); apotiris@med.uoa.gr (A.P.); theokarampitsakos@hotmail.com (T.K.); ctheofan@med.uoa.gr (C.T.); 4 Faculty of Medicine, Medical School, National and Kapodistrian University of Athens, 11528 Athens, Greece; isanagnostaki3@gmail.com; 5 First Department of Obstetrics and Gynecology, Alexandra Hospital, Medical School, National and Kapodistrian University of Athens, 11528 Athens, Greece; kdomali@yahoo.fr

**Keywords:** telomere length, preeclampsia, telomerase

## Abstract

**Background/Objectives**: Preeclampsia represents a significant obstetric complication, frequently linked to elevated levels of perinatal morbidity. This review sought to systematically examine the existing literature regarding associations between telomere length in maternal blood, placental tissue, and umbilical cord blood, and the occurrence of preeclampsia. **Methods**: A comprehensive search of PubMed/MEDLINE and ScienceDirect was conducted to identify studies published up to January 2025 that investigated telomere length in relation to preeclampsia. All observational studies comparing telomere length between women with preeclampsia and healthy pregnant controls were included. **Results**: A total of 838 studies were assessed. Although findings regarding the association between telomere length in leukocytes in maternal peripheral blood, placental tissue, and cord blood with preeclampsia remain inconsistent, the studies with the largest sample sizes for maternal blood and placental tissue have reported shorter telomere lengths in preeclamptic cases. In cases of preeclampsia, telomerase levels in leukocytes in maternal peripheral blood are elevated, whereas telomerase expression in placental tissue is reduced. **Conclusions**: Current evidence regarding the role of telomere length in preeclampsia remains inconsistent, precluding definitive conclusions. To clarify its potential as a biomarker, large-scale prospective studies are warranted to longitudinally assess telomere length in leukocytes in maternal peripheral blood, placental tissue, and cord blood, and to establish optimal threshold values.

## 1. Introduction

A primary objective in the field of medicine is to comprehend the etiologies of diseases. Preeclampsia (PE) and eclampsia, mysterious and elusive disorders associated with pregnancy, are classified among the “great obstetrical syndromes,” wherein a variety of often intersecting pathological processes converge to initiate a common pathway, culminating in the clinical identification of these disorders [1]. Significant risk factors encompass a history of PE, chronic hypertension, pregestational diabetes mellitus, antiphospholipid syndrome, and obesity, among other conditions [2]. Additional risk factors include advanced maternal age, nulliparity, a history of chronic kidney disease, and the use of assisted reproductive technologies (ARTs). Less common risk factors include a family history of PE and a mother carrying a fetus with trisomy 13 [3].

An increased rate of PE is associated with conceptions achieved through ARTs. The risk of PE is 1.71 times higher in women undergoing ART compared to those with spontaneous conception [4]. Recent observational studies indicate a correlation between the absence of the corpus luteum in artificial frozen embryo transfer (FET) cycles and an elevated risk of PE [5]. Compared to both in vitro fertilisation (IVF) with autologous oocytes and naturally conceived pregnancies, oocyte donation (OD) treatment is associated with a heightened risk of hypertensive disorders of pregnancy and PE. In particular, the risk of PE is two to three times higher in recipients of OD treatment compared to women undergoing IVF [6].

Telomeres are crucial structures composed of satellite DNA repeats situated at the termini of chromosomes in the majority of eukaryotic organisms [7]. Telomeric DNA consists of a repetitive sequence (GGTTAG/CCAATC in humans), predominantly double-stranded (ds; 10–15 kb in humans), but terminating in a short single-stranded (ss; 50–500 nt in humans) G-rich 3′ overhang [8]. Telomeres maintain genomic integrity by preventing the fusion of chromosomes. Telomere length (TL) varies within cells but typically follows a Gaussian distribution. The average length of the 92 telomeres in human leukocytes is a trait with a significant hereditary component [9]. TL subsequently decreases with age, generally at a steady rate [10]. TL can be influenced by a multitude of factors, including genetic predispositions, gender, ethnicity, levels of psychosocial stress, physical activity, obesity, smoking habits, and alcohol consumption [11]. However, the impact of telomeres on later life begins during pregnancy, with TL in newborns exerting a significant influence on health outcomes in adulthood [12,13,14].

Telomerase is a ribonucleoprotein reverse transcriptase that extends telomeric DNA to compensate for replication-associated telomere shortening. The enzyme consists of a catalytic subunit, telomerase reverse transcriptase (TERT), and an RNA component (TERC/TR), which provides the template sequence for telomere repeat addition. In human somatic cells, telomerase primarily limits progressive telomere shortening rather than directly mediating telomeric end protection, whereas in cancer cells it can contribute more directly to telomere capping and stabilization. Telomerase activity is not present in all proliferating cells, as certain cell types lacking detectable activity can undergo a finite number of population doublings before reaching replicative senescence. Most tissues are maintained by adult stem and progenitor cells that can upregulate telomerase activity when required. A defining feature of telomerase is its repeat addition processivity, which allows the enzyme to synthesize multiple telomeric repeats during a single binding event—an ability not observed in other known DNA or RNA polymerases [8,9,11].

The available data on TL in pregnant women with PE is limited and often contradictory. This systematic review and meta-analysis examine the impact of PE on TL in mothers.

## 2. Materials and Methods

### 2.1. Protocol, Data Sources and Search Strategy

This study adhered to the Preferred Reporting Items for Systematic Reviews and Meta-Analyses (PRISMA) checklist for conducting systematic reviews and meta-analyses [15]. The study protocol was registered on 12 September 2025, with registration number INPLASY202590043 in the International Platform of Registered Systematic Review and Meta-analysis Protocols (INPLASY, Middletown, DE, USA).

A detailed search strategy incorporating both MeSH terms and free-text keywords was designed for PubMed, Cochrane Library and suitably modified ScienceDirect. The search was conducted without any restrictions up to January 2025. The MeSH terms employed included “telomere,” “telomere length,” and “telomerase,” in combination with “preeclampsia” and “toxemia of pregnancy”. In addition, we manually examined the reference lists of the articles identified through this approach to locate further relevant studies. A search of the “grey literature” (e.g., medRxiv and the Grey Literature Report) was also conducted to identify other potentially eligible investigations.

### 2.2. Study Selection, Data Extraction and Quality Assessment

Two independent reviewers evaluated eligible studies by screening their titles and abstracts. Studies were included if they were original, peer-reviewed research that reported on maternal or offspring TL in women with PE and included a control group of normotensive pregnant women. Following the removal of duplicate entries and a preliminary screening of article titles and abstracts, studies that satisfied the inclusion criteria were subjected to a thorough review. Next, 43 full-text articles, identified as potentially pertinent, were scrutinized to compile the definitive list of included studies. Any disagreements among reviewers were resolved through discussion, with a third reviewer adjudicating as necessary. At this stage, 24 studies were excluded owing to the absence of comparative results between pregnant women with and without PE. Consequently, 19 studies were incorporated into the final analysis, examining maternal or offspring TL in pregnant women with and without PE.

**Figure 1:**
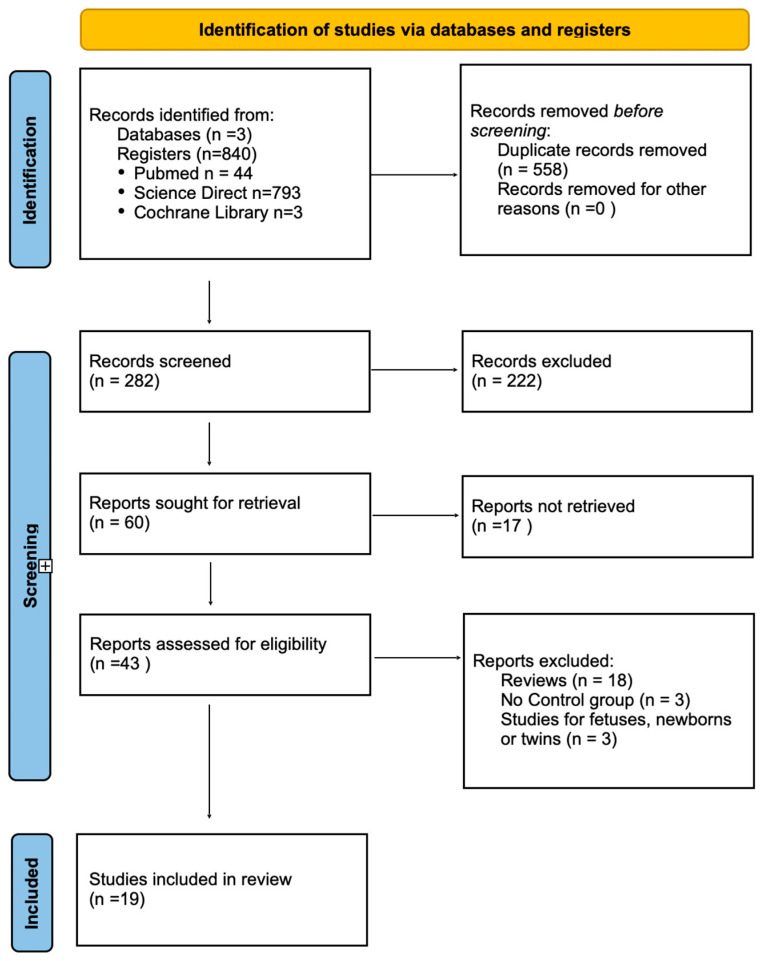
PRISMA flow diagram of the study selection process.

A quantitative meta-analysis was not pursued, as the included studies demonstrated substantial variability in their design, outcome definitions, measurement techniques, and population characteristics. Such heterogeneity would have undermined the reliability and interpretability of any pooled effect estimates. In addition, inconsistencies in reporting quality and the presence of potential confounders further limited the feasibility of generating a robust quantitative synthesis. For these reasons, we opted to present a qualitative evaluation of the evidence instead. Figure 1 illustrates the study selection process.

## 3. Results

### 3.1. Included Studies

Table 1, Table 2 and Table 3 provide details regarding the studies’ locations and durations, objectives, participant counts, age ranges, gestational ages, and methods utilized for telomere measurement measured in maternal blood, placental biopsies and cord blood.

Six studies were ultimately identified that assessed TL in maternal blood in relation to PE. A total of ten studies were detected that evaluated TL in placental biopsies in relation to PE. Four studies were revealed that investigated TL in cord blood in association with PE. Two studies documented telomerase activity, one in maternal blood and one in umbilical cord blood (Table 4).

### 3.2. Maternal Blood and Telomere Length

Findings from studies investigating TL in the blood of women with pre-eclampsia compared to healthy controls are inconsistent. Three studies reported no statistically significant difference, one study indicated longer telomeres in pre-eclamptic women, while two studies found shorter TL associated with PE.

Harville demonstrated that there is no difference in TL between healthy women and those with PE [16] while Han et al. demonstrated that a shorter TL was significantly associated with an increased risk of PE [17]. Zhang et al. reported that maternal relative TL was significantly longer in patients with PE compared to controls [18].

Pruszkowska-Przybylska et al. found that analyses of DNA methylation patterns indicate a potential reduction in TL among women with PE compared to those with normal blood pressure; however, this difference did not reach statistical significance [19]. Lekva et al. found no statistically significant differences in TL between the groups examined [20].

Panelli et al. observed a trend towards reduced leukocyte TL in women with PE within the Urban Cohort; however, this pattern was not evident in the Suburban Cohort. Further stratification by stress levels within the Urban Cohort indicated that PE was associated with shorter postpartum LTLs among women reporting moderate levels of stress [21].

### 3.3. Placental Biopsies and Telomere Length

Mandakh et al. reported no statistically significant association between placental relative TL and PE [22]. Yang et al. found that placental TL in cases of severe PE did not differ significantly from that of healthy controls. Instead, a strong inverse correlation was observed between gestational age and placental TL. The study also highlighted racial disparities in placental TL [23]. Placental TL was significantly longer in Latina mothers compared to non-Latina mothers (*p* = 0.009). Caucasian women with severe PE exhibited shorter placental TL than their non-Caucasian counterparts. These findings suggest that severe PE does not directly impact placental TL. Rather, TL is influenced by gestational age and maternal racial background [23].

Broady et al. demonstrated that TL did not differ significantly in cases of PE compared to controls [24]. A study by Sukenik-Halevy et al. found that telomere shortening in preeclamptic trophoblasts is associated with increased cellular senescence. Alterations in telomere maintenance mechanisms were observed in these cases. These findings support the involvement of telomeres in the pathogenesis of trophoblastic dysfunction in PE [25]. Wilson et al. provided evidence indicating that placental TL is not significantly affected by Early-Onset Preeclampsia (EOPE) or Late-Onset Preeclampsia (LOPE) [26].

Farladansky-Gershnabel et al. found that the percentage of trophoblasts with short telomeres was higher in placental samples from EOPE compared to LOPE, with both conditions showing higher levels than controls. Additionally, aggregate formation was more pronounced in EOPE compared to LOPE, with both showing higher levels than in healthy controls [27]. Telomere aggregates are conglomerates of telomeric regions that arise predominantly under conditions of cellular stress or during senescence, forming three-dimensional clustered configurations within the nucleus. Their occurrence is independent of both telomere length and telomerase activity [27].

Baser et al. demonstrated that telomeres are shorter in pregnant women suffering from PE compared to those with uncomplicated pregnancies [28]. The study by Biron-Shental et al. found that TL is reduced in pregnant women with PE, regardless of whether the fetus is affected by fetal growth restriction (FGR), compared to women with uncomplicated pregnancies [29].

Cecati et al. demonstrated a 40% reduction in TL in placental samples from women with PE compared to healthy controls [30] while Manna et al. reported higher telomere lengths in women with preeclampsia, although the difference was not statistically significant [31].

### 3.4. Cord Blood and Telomere Length

Verner et al., in the PREDO cohort study, found no statistically significant association between TL in cord blood and PE [32]. Furthermore, Sukenik-Halevy et al. reported no significant differences in TL in leukocytes isolated from cord blood cells when comparing pregnancies affected by pre-eclampsia to those in the control group. Similarly, the proportion of aggregates did not differ markedly between the two cohorts [25].

Baser et al. reported that telomere length in cord blood of newborns with pre-eclampsia was significantly reduced compared with controls. Both total antioxidant status (TAS) and total oxidant status (TOS) were elevated in cord blood and placental tissue (*p* < 0.05). Multivariate logistic regression identified placental TOS levels as an independent risk factor for pre-eclampsia (OR = 1.212, 95% CI = 1.068–1.375). While TAS was also increased, this may reflect a compensatory response to higher TOS, as antioxidants rise to counteract elevated oxidative stress. Therefore, the observed telomere shortening is more likely associated with elevated TOS, indicating increased oxidative stress, rather than higher antioxidant levels [28].

Zhang et al. observed that the relative TL in cord blood was significantly greater in pre-eclamptic patients than in the control group (median: 0.61 vs. 0.35; *p* < 0.001) [18].

### 3.5. Telomerase Activity

Madendag et al. reported a correlation between **TERT protein** levels and pre-eclampsia severity, with levels of 1.137 ± 0.390 ng/mL in the severe group, 0.763 ± 0.390 ng/mL in the non-severe group, and 0.425 ± 0.160 ng/mL in controls (*p* < 0.001) [33].

Hwang et al. reported a significant reduction—approximately 40%—in telomerase activity within mesenchymal stem cells isolated from human umbilical cord blood (hUCB-MSCs) of preeclamptic pregnancies, relative to those obtained from pregnancies without hypertensive complications [34].

### 3.6. Risk of Bias Assessment

A formal quality assessment using the Newcastle–Ottawa Scale was performed for all 9 included studies. The majority were found to have moderate to low risk of bias. The overall quality of evidence was deemed sufficient to support the review’s conclusions. A full summary of NOS scoring is provided in Table 5. For selection, four criteria were used: (a) Case definition adequacy (1★), (b) Representativeness of cases (1★), (c) Selection of controls (1★) and Definition of controls (1★). For Comparability, one criterion was adjusted for key confounders (e.g., age, BMI, gestational age) while for Outcome/Exposure, three criteria were implemented: (a) Exposure/outcome assessment (1★), (b) Same method for cases/controls (1★) and non-response rate (1★). Each study is scored out of 9 stars (★), with higher scores indicating lower risk of bias.

To enhance methodological transparency, Table 5 includes a separate column specifying the TL and telomerase-related parameter assessed levels methods for each study. TL was measured by Southern blot, qPCR, or FISH-based methods all of which are considered high-quality and precise for either population-level or single-cell TL assessment. Telomerase activity was measured directly by TRAP assay, whereas a study measured TERT protein only, which does not reflect enzymatic activity. Studies using high-quality TL and TA measurements were considered at low risk of bias for Outcome/Exposure, while studies with indirect or insufficiently described methods were considered to have a moderate risk of bias. No additional stars were assigned for TL/TA methods; instead, their quality and limitations were considered in the narrative justification for the Outcome/Exposure domain, preserving the standard NOS scoring framework.

In summary, 11 studies had low risk of bias (7–9★), 7 studies had moderate risk of bias (5–6★) while no studies showed high risk of bias (≤4★). Table 5 includes the results of the risk of bias assessment of the present review.

## 4. Discussion

This systematic review explores the association between TL in maternal blood, placental tissue, and umbilical cord blood, as well as telomerase levels, aiming to highlight potential correlations.

Telomeres, located at the ends of linear eukaryotic chromosomes, are vital for genomic stability and typically consist of double-stranded guanine-rich tandem repeats with a single-stranded G-rich 3′ overhang [35].

TL is influenced by both maternal and paternal genetic components [36,37]. Factors such as oxidative stress, biological ageing, metabolic conditions, diabetes, increasing age, elevated BMI, and geographical location have all been associated with a reduction in TL [38,39].

Telomerase is a specialised DNA polymerase that extends the 3′ ends of chromosomes by processively synthesising multiple telomeric repeats, functioning as a unique ribonucleoprotein complex composed of telomerase reverse transcriptase (TERT) and an RNA component (TER) that provides the template for synthesis [40,41]. Telomerase activity, regulated in a spatiotemporal manner, is crucial for embryonic and placental development. Its association with maternal age, fertilisation success, early embryonic processes, and pregnancy complications such as Fetal Growth Restriction (FGR) and fetal hypoxia highlights its significance in reproductive outcomes [42].

Εxisting studies have yielded conflicting findings regarding TL in maternal blood and its potential association with PE [16,17,18,19,20,21]. This inconsistency may be attributed to a lack of stratification to control for confounding variables, as well as significant heterogeneity among studies in terms of maternal age, gestational age, and coexisting conditions such as gestational diabetes. In a study reporting increased telomere length, a plausible explanation can be advanced. Higher levels of physical activity among participants with PE may have contributed to these unexpected findings. Although they began pregnancy with abnormal clinical profiles, most women adhered closely to medical recommendations such as daily folate intake and maintained generally healthy lifestyles—factors that are well established to correlate with longer leukocyte telomeres and attenuated telomere shortening. Such behavioral changes may therefore have helped to preserve telomere length in mild and well-managed PE cases [18].

However, the study with the largest sample size reported that TL in maternal blood was significantly shorter among women with PE compared to healthy pregnant controls [17].

Similarly, research investigating placental TL in relation to the development of PE has produced inconclusive findings. The evidence remains inconsistent, with some studies reporting shortened telomeres in preeclamptic placentas, while others have found no significant association [22,23,24,25,26,27,28,29,30,31]. Notably, the study by Yang et al., which included the largest sample size to date, demonstrated no difference in TL between patients with severe PE and healthy controls [23]. The authors suggested that TL may instead be influenced by maternal age and racial background. This study reports, for the first time, longer telomere lengths in placentas from Latino mothers, particularly those affected by severe preeclampsia [23].

The existing literature presents conflicting evidence regarding TL measured in umbilical cord blood and its potential association with PE. These inconsistencies may be influenced by various contributing factors, including maternal age, gestational age, and BMI [18,25,28,32].

With regard to **TERT protein** levels in maternal plasma, Madendag et al. demonstrated that these levels are elevated in cases of PE, with the degree of increase correlating with the severity of the condition. A key strength of this study lies in its efforts to control for potential confounders; variables such as maternal age, BMI, gestational age at the time of blood sampling, smoking history, ethnicity, and history of caesarean section were statistically comparable across the study groups [33].

Ultimately, a study conducted by Hwang et al. revealed that telomerase activity in mesenchymal stem cells derived from umbilical cord blood was approximately 40% lower in women diagnosed with PE compared to those with uncomplicated pregnancies [34].

Dysfunctional telomeres can form aggregates that disrupt chromosome segregation, causing genetic instability, apoptosis, and senescence, which in turn may contribute to chronic disease development and impaired tissue function [43,44]. Telomere aggregate formation is a stress-driven phenomenon that is not contingent upon telomere length or the activity of telomerase [29]. This may help explain why telomere length findings in preeclampsia are sometimes inconsistent. Such mechanisms may underlie the observed variability in telomere length reported in preeclampsia.

Nonetheless, the interpretation of findings is substantially constrained by considerable heterogeneity across studies. Notable variations were observed in participant selection criteria and in the methodologies employed to measure TL and telomerase activity. In addition, numerous studies presented their findings using medians, interquartile ranges, or logarithmic transformations, further complicating comparisons. A further limitation stems from the frequent omission of adjustments for potential confounding variables. Factors such as maternal age, gestational age, BMI, gestational diabetes mellitus, stage of pregnancy, smoking status, and parity are all known to influence telomere dynamics but were often not adequately accounted for.

Future well-designed studies are essential to clarify the potential association between TL—in maternal tissues, placental samples, and umbilical cord blood—and the development of PE. Such investigations should incorporate appropriate stratification for potential confounding variables, including maternal age, gestational age at the time of sample collection, the employment of ARTs, lifestyle factors, nutritional status, ethnicity, and the presence of pre-existing or coexisting conditions such as autoimmune diseases, gestational diabetes, or FGR, as well as environmental exposures [45,46]. These studies should incorporate measurements of telomere aggregates and SIRT1 in order to provide the most accurate and informative results possible. Moreover, it is important to recognize that an observed association does not imply a causal relationship, particularly given the inherent limitations of observational study designs, which preclude the establishment of causality [11]. Even in cases where telomere shortening is detected in patients with PE, it remains unclear whether telomere attrition serves as a contributing factor, a consequence of the disease process, or both [11].

## 5. Conclusions

The available evidence indicates that telomere length may be reduced in leukocytes in maternal peripheral blood, placental tissue, and offspring (umbilical cord blood) in the context of preeclampsia. However, several of the included studies were assessed as having a moderate risk of bias, and variability in study design, populations, and measurement methods limits the strength of the conclusions. Therefore, these findings should be interpreted with caution, and further high-quality, well-controlled studies are needed to confirm and clarify the observed associations.

## Figures and Tables

**Table 1:** Maternal blood and telomere length.

Authors	Location and Period of Study	Sample Size and Maternal Age	Gestational Age	BMI or Weight Gain	Telomere Measurement	Telomere Length (Mean ± SEM) or Results
[16]	Seattle & Tacoma, Washington, USA. April 1998 to June 2002.	PE *n* = 25; age: 27.3 ± 0.6. Control women, *n* = 50; age: 29.5 ± 0.5.	PE 34.4 ± 0.6 Control 39.7 ± 0.2	Pre-pregnancy BMI: PE 26.9 ± 7.5; control group, 22.4 ± 0.4.	quantitative PCR, leukocytes in maternal peripheral blood	PE 0.77 ± 0.02 Control 0.77 ± 0.02
[17]	The TL summary statistics were derived from the largest GWAS on TL (dataset ID: ieu-b-4879), analysing 472,174 European UK Biobank participants	PE *n* = 3903 controls *n* = 114,735	n/a	n/a	qPCR analysis, leukocytes in maternal peripheral blood	The IVW model indicated a nominally significant association between TL and PE (OR = 0.799; 95% CI = 0.651–0.979; *p* = 0.031).
[18]	Cases of preeclampsia and control participants were recruited from Nanjing Drum Tower Hospital, located in Nanjing, Jiangsu Province, Eastern China, during the period from January 2019 to June 2020.	PE *n* = 130 age < 25, *n* = 14 age: 25–29, *n* = 56 age: 30–34, *n* = 38 age ≥ 35, *n* = 22 Controls *n* = 341 age < 25, *n* = 27 age: 25–29, *n* = 179 age: 30–34, *n* = 104 age ≥ 35, *n* = 31	PE GA: 37.20 ± 0.28 Control GA: 39.19 ± 0.13	BMI at delivery PE: Quantile 1: *n* = 30 Quantile 2: *n* = 20 Quantile 3: *n* = 30 Quantile 4: *n* = 50 Controls Quantile 1: *n* = 89 Quantile 2: *n* = 96 Quantile 3: *n* = 87 Quantile 4: *n* = 67	modified qPCR, leukocytes in maternal peripheral blood	Maternal relative TL was significantly longer in PE patients compared to controls (median: 0.48 vs. 0.30; *p* < 0.001).
[19]	Royal Women’s Hospital (RWH), Melbourne, Australia 2007 to 2011.	PE *n* = 77 age: 30.53 ± 5.68 Controls *n* = 89 age: 31.76 ± 4.96	n/a	n/a	Qiagen’s Blood and Cell Culture DNA Midi Kit (Venlo, The Netherlands)	TL may be lower in women with PE compared to normotensive women, though the difference was not significant.
[20]	Oslo University Hospital Rikshospitalet, Oslo, Norway, between 2002 and 2008	PE *n* = 31 age: 30 ± 4 Controls *n* = 186 age: 32 ± 4	enrolment at 22–24 weeks of gestation	BMI at 22–24 weeks PE: 29.0 (23.8, 32.1) Controls: 24.8 (22.5, 26.8)	RNA was extracted using the Magnapure Isolation Kit (Roche, Basel, Switzerland) and instrument for samples collected at weeks 22–24, and the MagMAX Isolation Kit (Roche, Basel, Switzerland) and instrument for those collected at weeks 36–38.	No significant differences in TL were observed
		PE *n* = 34 age: 32 ± 6 Controls *n* = 61 age: 32 ± 5	PE GA: 35 ± 4 Controls GA: 36 ± 0	BMI at blood sampling PE: 30.1 (25.8, 34.2) Controls: 27.3 (24.6, 28.9)		
[21]	The Suburban Cohort was conducted by the Prematurity Research Center at a prominent academic institution on the West Coast, spanning from 2012 to 2018.	Median PE *n* = 21 age: 36 (6) Controls *n* = 33 age: 30 (5)	Enrolment prior to 20th week of gestation	Median BMI at enrolment PE 26.6 (11.2) Controls 23.1 (6.7)	TL measured by qPCR	In the Urban Cohort, women with preeclampsia tended to have shorter leukocyte TLs (6517 vs. 6913 bp; *p* = 0.07), a pattern not seen in the Suburban Cohort. Among those with moderate stress, PE was linked to significantly shorter postpartum LTLs (*p* = 0.02)
	The Urban Cohort conducted by a prospective birth cohort at an East Coast academic institution, spanning from 2000 to 2012.	Median PE *n* = 30 age: 29 (10) Controls *n* = 30 age: 29 (9)	Median First postpartum day PE GA: 36.0 (3) Controls GA: 39.5 (2)	Median PE 30.6 (6.3) Controls 24.6 (6.2)		

**Table 2:** Placenta biopsies and telomere length.

Authors	Location and Period of Study	Sample Size and Maternal Age	Gestational Age	BMI or Weight Gain	Telomere Measurement	Telomere Length (Mean ± SEM) or Conclusion
[22]	Skåne University Hospital in Lund and Malmö, Sweden. 2008 to 2015	PE *n* = 42; age: 30.5 ± 5.9. Normotensive women, *n* = 95; age: 29.97 ± 4.1.	PE 269.3 ± 14.6 days, Normotensive 276.3 ± 15.9	Pregestational BMI in PE 27.6 ± 6.3 Pregestational BMI in Normotensive women 24.9 ± 4.5	TL measured by qPCR	PE 1.36 ± 0.33 Control 1.41 ± 0.38
[23]	Kapiolani Women and Children’s Hospital, Hawaii, USA January 2006 to June 2013	PE *n* = 95; age: 29.87 (SD = 6.64) Normotensive women, *n* = 129; age: 29.53 (SD = 6.53)	PE 35.28 (SD = 3.01) Normotensive 39.33 (SD = 0.85)	Pregestational BMI in PE 28.53 (SD = 7.66) Pregestational BMI IN Normotensive women 28.01 (SD = 6.18)	TL measured by qPCR	ln-transformed placental TL PE 5.53 (SD = 0.43) Control 5.41 (SD = 0.38)
[24]	Kapi’olani Medical Center for Women and Children (Honolulu, HI), 2013–2014	PE but without SGA (*n* = 18) Gestational-age matched controls (*n* = 16)	n/a	n/a	TL measured by Southern blot	No significant difference in TL
[25]	Meir Medical Center, Kfar Saba, Israel	PE without FGR *n* = 9, age: 33.1 s = 6.8 Controls *n* = 14, age: 28.8 s = 5.2	PE 35.9 SD = 3.2 Uncomplicated pregnancies 39 SD = 1	Average BMI (kg/m^2^) 23.1 SD = 3.3 22.6 SD = 3.6	TL and telomeric aggregates in cord blood cells assessed by qFISH	Telomere shortening in trophoblasts from pregnancies complicated with PE were observed.
[26]	Department of Medical Genetics, University of British Columbia, Canada	EOPE *n* = 21 LOPE *n* = 18 Controls > 24 wks *n* = 59	EOPE 24.9–37.6 wks (F: 8, M: 13) LOPE 34.6–41.3 wks (F: 9, M: 9) Controls 25.7–40.3 wks (F: 27, M: 32)	n/a	Placental TL measured by qPCR	Male fetuses exhibited shorter TL. Maternal age showed no significant relationship with TL, nor did fetal birth weight.
[27]	Department of Obstetrics and Gynecology, Meir Medical Center, Kfar Saba, Israel	EOPE *n* = 7 age: 32.8 (SD = 6.5) gestational age-matched healthy controls *n* = 7 age: 29 (7.4) LOPE *n* = 6 age: 35.25 (SD = 5.5) gestational age-matched healthy controls *n* = 6 age: 27.8 (SD = 3.6)	EOPE 32.3 (SD = 2.7) LOPE 37 (SD = 1.1)	BMI (at term) (SD) EOPE: 32 (SD = 5.6) Control: 31.5 (SD = 8.2) LOPE: 31 (SD = 0) Control: 25.5 (SD = 1.2)	Placental TL and telomeric aggregates assessed by qFISH	Short telomeres were observed in EOPE cases (compared to LOPE cases (28.72% ± 10.14%), with both groups displaying markedly higher percentages than the controls (7.53% ± 5.14%, *p* = 0.03).
[28]	Department of Obstetrics and Gynecology, Yozgat Bozok University, Yozgat, Turkey	PE *n* = 27 healthy controls *n* = 53	PE GA: 28 wks–33 wks and 6 days	-	TL in placenta measured by qPCR	TLs of patients with PE were significantly shorter compared to those of the control group.
[29]	Departments of Obstetrics and Gynecology and Pathology and the Genetic Institute, Meir Medical Center, Kfar Saba; the Faculty of Life Sciences, Bar Ilan University, Ramat Gan and Sackler School of Medicine, Tel Aviv University, Tel Aviv, Israel.	PE without FGR *n* = 14 FGR without PE *n* = 14 PE and FGR *n* = 9 Gestational Age-matched Controls *n* = 20	PE without FGR GA: 36 ± 1.41 FGR without PE GA: 36 ± 2.43 PE and FGR GA: 36.2 ± 3.5 Gestational Age-matched Controls GA: 37.15 ± 3.9	n/a	FISH	Telomeres are shorter in placental trophoblasts from pregnancies with PE, FGR, and both PE and FGR, compared to trophoblasts from uncomplicated pregnancies
[30]	Patients recruited from the Department of Clinical Sciences at Università Politecnica delle Marche, Ancona, Italy.	PE *n* = 34 age: 30.2 ± 1.3 Controls *n* = 34 age: 31.5 ± 0.3	PE GA: 31.2 ± 1.1 Controls GA: 32.5 ± 3.5	n/a	Placental TL measured by qPCR	A marked 40% reduction in TL n tissues obtained from pathological subjects
[31]	Cork University Maternity Hospital, Ireland, 2019 and 2021.	PE *n* = 5 age: 38.6 ± 5.2 PE and FGR *n* = 8 age: 33.35 ± 3.1 FGR *n* = 6 age: 33.67 ± 3.1 Term Controls *n* = 20 age: 33.7 ± 4.40	PE GA: 36 ± 1.9 (weeks ± SD) PE and FGR GA: 34.6 ± 2.7 FGR GA: 32.43 ± 13.1 Term Controls GA: 38 ± 0.7	PE: 29.9 ± 9.6 PE and FGR: 27.7 ± 3.7 FGR: 25.31 ± 3.5 Term Controls 24.4 ± 5.4	TL measured by qPCR	Placental absolute TL was found to be longer in PE and in PE associated with FGR compared to controls. Not statistically significant.

**Table 3:** Cord blood and telomere length.

Authors	Location and Period of Study	Sample Size and Maternal Age	Gestational Age	BMI or Weight Gain	Telomere Measurement	Telomere Length (Mean ± SEM) or Results
[32]	PREDO Cohort Study, Finland. 2006 to 2010.	Total Sample *n* = 602, 33.2 (SD = 5.48) PE *n* = 47	39.73 (SD = 1.71)	pre-pregnancy BMI = 26.97 (SD = 6.31)	qPCR in leukocytes extracted from cord blood	N/A
[25]	Meir Medical Center, Kfar Saba, Israel	PE without FGR *n* = 9, age: 33.1 s = 6.8 Controls *n* = 14, age: 28.8 s = 5.2	PE 35.9 SD = 3.2 Uncomplicated pregnancies 39 SD = 1	Average BMI (kg/m^2^) 23.1 SD = 3.3 22.6 SD = 3.6	FISH was conducted to assess TERC copy number, telomere capture, and SAHF in both trophoblasts and cord blood cells	No significant differences in TL and in the proportion of aggregates
[28]	Department of Obstetrics and Gynecology, Yozgat Bozok University, Yozgat, Turkey	PE *n* = 27 healthy controls *n* = 53	PE GA: 28 wks–33 wks and 6 days	-	TL in cord blood cells measured by qPCR	TL in patients with PE were significantly shorter
[18]	Nanjing Drum Tower Hospital, located in Nanjing, Jiangsu Province, Eastern China, during January 2019 to June 2020.	PE *n* = 130 age < 25, *n* = 14 age: 25–29, *n* = 56 age: 30–34, *n* = 38 age ≥ 35, *n* = 22 Controls *n* = 341 age < 25, *n* = 27 age: 25–29, *n* = 179 age: 30–34, *n* = 104 age ≥ 35, *n* = 31	PE GA: 37.20 ± 0.28 Control GA: 39.19 ± 0.13	BMI at delivery PE: Quantile 1: *n* = 30 Quantile 2: *n* = 20 Quantile 3: *n* = 30 Quantile 4: *n* = 50 Controls Quantile 1: *n* = 89 Quantile 2: *n* = 96 Quantile 3: *n* = 87 Quantile 4: *n* = 67	Genomic DNA was extracted from leukocytes in cord blood, with TL and mtDNA-CN analysed using modified qPCR	RTL in cord blood was significantly longer in PE patients

**Table 4:** Telomerase levels.

Authors	Location and Period of Study	Sample Size and Maternal Age	Gestational Age at Delivery	BMI or Weight Gain	Telomerase-Related Parameter Assessed Measurement	Telomerase-Related Parameter Assessed Levels
[33]	Erciyes University, The Faculty of Medicine, the Perinatology Clinic, Turkey	Severe PE group *n* = 28, age: 28.6 ± 2.1 Non-severe PE group *n* = 28 age: 29.1 ± 2.3 Control group *n* = 30 29.4 ± 2.6	Severe PE group 33.6 ± 2.1 Non-severe PE group 37.1 ± 1.7 Control group 39.6 ± 1.5	Body mass index (BMI) at blood sampling (kg/m^2^) Severe PE group 30.2 ± 4.4 Non-severe PE group 30.7 ± 3.4 Control group 30.1 ± 4.1	TERT protein measured by ELISA	TERT protein levels were higher in severe and non-severe PE than in controls (*p* < 0.001)
[34]	Konkuk University Medical Center, Konkuk University School of Medicine, Seoul, Republic of Korea	PE *n* = 28 32 ± 2.5 Normal *n* = 30 31 ± 2.1	PE 35 ± 1.7 Normal 36 ± 1.6	N/A	Telomerase activity measured directly after isolation using conventional TRAP assay (Roche)	Telomerase activity in hUCB-MSCs was found to be lower in PE.

**Table 5:** Risk of Bias Assessment (Newcastle–Ottawa Scale) with Justifications.

Study	Selection (Max 4★)	Comparability (Max 2★)	Outcome/Exposure (Max 3★)	TL/TA Method	Total Score (9★)	Risk of Bias	Key Characteristics Justifying Score
[16]	★★★★	★★	★★★	quantitative PCR	9	Low	- Clear case/control definitions. - Adjusted for age, BMI, and gestational age. - Used standardized TL measurement (qPCR).
[17]	★★★	★★	★★★	qPCR analysis	8	Low	- Large sample size (GWAS data). - Adjusted for genetic confounding. - Limited details on control selection.
[18]	★★★★	★★	★★★	modified qPCR	9	Low	- Matched cases/controls by age and BMI. - Blinded TL measurement. - Longitudinal design.
[19]	★★★	★★	★★	Qiagen’s Blood and Cell Culture DNA Midi Kit (Qiagen, Venlo, The Netherlands)	7	Moderate	- Small sample size. - Adjusted for age but not parity/smoking. - Used methylation proxies for TL (indirect measure).
[20]	★★★★	★★	★★★	NA was extracted using the Magnapure Isolation Kit and the MagMAX Isolation Kit and instrument (Roche, Basel, Switzerland)	9	Low	- Population-based cohort. - Adjusted for key confounders. - Validated TL assay (qPCR).
[21]	★★★★	★★	★★★	TL measured by qPCR	9	Low	- Multicohort design. - Stratified by stress levels. - Controlled for ethnicity and SES.
[22]	★★★	★★	★★	TL measured by qPCR	7	Moderate	- Placental TL only. - No adjustment for pollution exposure timing. - Small control group.
[23]	★★★★	★★	★★★	qPCR	9	Low	- Large sample (*n* = 95 cases). - Adjusted for race/gestational age. - Robust FISHbased TL measurement.
[24]	★★★	★★	★★	Southern blot	7	Moderate	- No adjustment for placental pathologies. - Small sample (*n* = 18 PE cases). - Used Southern blot low measurement bias method
[25]	★★★★	★★	★★★	FISH	9	Low	- Matched for gestational age. - Blinded FISH analysis. - Controlled for fetal sex.
[26]	★★★	★★	★★	Placental TL measured by qPCR	7	Moderate	- Focused on EOPE/LOPE differences. - Unadjusted for maternal comorbidities. - Small subgroups.
[27]	★★★★	★★	★★★	Placental TL and telomeric aggregates assessed by qFISH	9	Low	- Clear EOPE vs. LOPE stratification. - Blinded scoring.
[28]	★★★	★★	★★	TL in placenta and cord blood cells measured by qPCR	7	Moderate	- Small sample (*n* = 34 PE). - Adjusted for oxidative stress but not BMI. - Singlec-enter design.
[29]	★★★★	★★	★★★	FISH	9	Low	- Matched for FGR status. - Controlled for placental pathology. - Used dual TL assays (qPCR/FISH).
[30]	★★★	★★	★★	Placental TL measured by qPCR	7	Moderate	- Small control group (*n* = 20). - Single timepoint sampling.
[31]	★★★★	★★	★★★	TL measured by qPCR	9	Low	- Adjusted for IUGR confounders. - Long-term control group (*n* = 20).
[32]	★★★	★★	★★	qPCR in leukocytes extracted from cord blood	7	Moderate	- Cohort study but lacked PE severity stratification. - Unadjusted for maternal stress. - Cord blood TL only.
[33]	★★★★	★★	★★★	TERT protein measured by ELISA	9	Low	- Stratified by PE severity. - Adjusted for maternal age/BMI. - indirect measurement method→ potential limitation
[34]	★★★	★★	★★	Telomerase activity measured directly after isolation using conventional TRAP assay (Roche)	7	Moderate	- Small sample (*n* = 28 PE). - No adjustment for stem cell passage number. - Single lab protocol. - High-quality measurement

## Data Availability

No new data were created or analyzed in this study.

## Abbreviations

The following abbreviations are used in this manuscript:

PE	Preeclampsia
TL	Telomere Length
RTL	Relative Telomere Length
ART	Assisted Reproduction Technique
FET	Frozen Embryo Transfer
IVF	In Vitro Fertilization
OD	Oocyte Donation
PRISMA	Preferred Reporting Items for Systematic Reviews and Meta-Analyses
ICD-10	10th Revision of the International Statistical Classification of Diseases and Related Health Problems
EOPE	Early-Onset Preeclampsia
LOPE	Late-Onset Preeclampsia
FISH	Fluorescence In Situ Hybridization
SAHF	Senescence-Associated Heterochromatic Foci
hUCB-MSCs	Human Umbilical Cord Blood-Derived Mesenchymal Stem Cells
FGR	Fetal Growth Restriction
BMI	Body Mass Index
TERT	Telomerase Reverse Transcriptase
TER	Telomerase RNA Component
GWAS	Genome-Wide Association Study
qPCR	Quantitative Polymerase Chain Reaction
ELISA	Enzyme-Linked Immunosorbent Assay
TAS	Total Antioxidant Status
TOS	Total Oxidant Status
GA	Gestational Age
SBP	Systolic Blood Pressure
DBP	Diastolic Blood Pressure
OR	Odds Ratio
CI	Confidence Interval
SEM	Standard Error of the Mean
SD	Standard Deviation
SIRT1	Sirtuin-1
eNOS	Endothelial Nitric Oxide Synthase
